# Barriers to Blood Pressure Control in Treated Patients Attending Public Primary Healthcare Clinics in Gaborone, Botswana

**DOI:** 10.1002/puh2.70124

**Published:** 2025-09-30

**Authors:** Setlhare V, Bogatsu Y, Youssouf N, Mwita J

**Affiliations:** ^1^ Department of Family Medicine and Public Health Medicine University of Botswana Gaborone Botswana; ^2^ London School of Hygiene and Tropical Medicine London UK; ^3^ Department of Internal Medicine University of Botswana Gaborone Botswana

**Keywords:** barriers, primary healthcare, public clinics, uncontrolled blood pressure

## Abstract

**Background:**

High blood pressure (BP) is prevalent and uncontrolled in many low‐ and middle‐income countries (LMICs). Uncontrolled BP causes cardiovascular diseases (CVDs) and kidney disease. The high morbidity and mortality caused by uncontrolled BP results in high health expenditures for families and governments. Knowledge of the barriers to BP control may help in crafting suitable interventions. The aim of this study was to explore the barriers to BP control among patients who were on treatment for high BP in public clinics in Gaborone, Botswana.

**Methods:**

This was a descriptive qualitative study to explore the barriers to BP control among patients already on BP treatment in public primary healthcare (PHC) clinics in Gaborone, Botswana. Data were collected from patients, nurses, doctors and district health managers, through structured interviews. The interviews were conducted using an interview guide, and they were audio recorded. The interviews were translated into English transcripts, and the transcripts were analysed for themes using NVivo, a software that is used in qualitative data analysis.

**Results:**

Eighteen participants were interviewed (six patients, four nurses, four doctors and four district health managers). The barriers to BP control were health system‐related (e.g., drug and equipment shortages and poor clinic administration), healthcare worker (HCW)‐related (e.g., poor attitudes and lack of knowledge), patient‐related (e.g., non‐adherence to treatment and negative lifestyle habits) and other socioeconomic factors.

**Conclusions:**

Barriers to BP control among patients receiving BP treatment were multifactorial and included health system, HCW, patient, and socioeconomic factors. There needs to be a holistic approach to overcome these barriers, and interventions should be aimed at central government funding and planning of health services, district‐level management of health services, proper management and resourcing of clinics, as well as HCW and patient education.

## Graphical abstract

Barriers to BP control among patients who are being treated for high BP expose patients to the risk of death, morbidity and health costs. These barriers are related to health system, healthcare worker, patient, lifestyle and socioeconomic factors. Knowledge of context‐specific barriers may help craft appropriate remedial interventions.

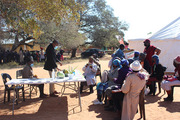

## 1 Background

High blood pressure (BP) is a major cause of premature death, a major risk factor for cardiovascular disease, and is prevalent worldwide; 25% of men and 20% of women have high BP [1, 2]. This translates to 1.28 billion people having high BP worldwide, and 67% of them live in low‐ and middle‐income countries (LMICs) [3].

By 2025, 1.5 billion people worldwide will be suffering from high BP, and 10.4 million will die annually from hypertension‐related deaths [2]. High BP contributes to 12.8%–13.5% of deaths worldwide, 54% of strokes and 47% of coronary heart disease [4, 5]. Worldwide, 8.5 million deaths in 2015 were attributable to high BP, and 88% of these deaths were in LMICs [6].

Among high BP patients aged 35–84 years, those with controlled BP were 59%, 32% and 25% in the United States, England and Japan, respectively [7]. Treatment coverage and BP control have increased in some high‐income countries (HICs) to around 80% and 70%, respectively [8]. However, high BP is poorly controlled in sub‐Saharan Africa, and poorly controlled BP is the commonest risk factor for cardiovascular disease [9]. Some studies have shown that many patients in Botswana (at least 40%) still have high BP despite being on treatment [10, 11, 12, 13]. High BP in Botswana is defined as BP above 140/90 [14, 15], and BP remains high in most BP patients in Botswana despite free BP treatment in the public healthcare sector. Medication adherence in this study, and in the Botswana context, is defined as taking medications as prescribed by a healthcare worker (HCW), and adherence is affected by patients’ ability to report for regular medical reviews at appointed times.

Effective, evidence‐based interventions (EBIs) for prevention and control of high BP exist and should prevent the high morbidity and mortality from the complications of high BP (strokes, ischaemic heart and chronic kidney disease) [1, 16]. These EBIs range from prevention strategies to lifestyle modification and to pharmaceutical interventions [17, 18]. Most BP patients should have normal BP with these evidence‐based BP control interventions. However, as with other EBIs, high BP EBIs do not always translate into BP control. Various barriers contribute to the failure to get expected results from implementing high BP EBIs.

These barriers include lack of screening for hypertension, alcohol abuse, smoking, obesity, high salt intake, low potassium intake, inactivity, gender, age, religion, diabetes and non‐adherence to treatment [19, 20, 21]. The barriers also comprise challenges in balancing appropriate use and adaptation of the EBIs, deficient monitoring of implementation processes, client factors, health service provider factors, innovation, structural factors, societaland budget factors [22, 23].

Barriers to BP control among treated patients have been known for a long time, and they are patient‐related (e.g., lack of adherence to treatment, detrimental lifestyle habits and comorbidities), physician‐related (e.g., treatment inertia, doctor–patient interaction and lack of knowledge) and health system‐related (e.g., lack of access to care, cost of drugs and poverty) [24]. Such barriers inhibit the successful implementation of high BP EBIs. Low health funding, shortage of HCWs, shortage of medicines, shortage of equipment, long queues in clinics, HCW attitudes and lack of adherence to treatment, as cited above, may compromise EBI implementation. It is, therefore, necessary to craft and implement effective strategies to achieve more success in controlling high BP worldwide, including in Botswana.

Most people with high BP are unaware that they have the disease, and only 21% have their hypertension under control. In China, only 6.1% of patients on treatment for high BP had controlled BP [7]. Many LMICs have BP control rates of 10%, whereas some HICs have control rates of 20% [6].

As a result, high BP is a significant cause of premature death, and one of the WHO global targets for NCDs is to reduce hypertension prevalence by 33% by the end of 2030 [3]. However, studies show that though the World Health Organisation aims to reduce the 2010 prevalence rate of high BP by 25% by 2025, trends show that this will not be achieved in sub‐Saharan Africa [25]. One study in Botswana showed that 63% of patients on treatment for high BP are poorly controlled, and this lack of BP control was associated with being male, being obese and not receiving lifestyle advice [15]. This lack of BP control in the Botswana public primary healthcare (PHC) clinics occurs despite provision of free medical care in the public health sector of Botswana.

In a study in Sudan, 54.7% of treated BP patients were not controlled, and non‐adherence to medication in 30% of treated patients was the main reason for lack of control [26]. In Thailand, 26% of treated BP patients were uncontrolled, and the main barriers to BP control included being male, diabetic, being obese and taking many BP drugs [20].

The low BP control rates among treated patients in Botswana are concerning and put many patients at risk of early death. This study aimed at exploring the barriers of BP control among patients who were on BP treatment in public clinics in Gaborone, Botswana. Knowing the barriers of BP control in Gaborone, Botswana, is likely to help craft remedial interventions and save lives.

We chose the qualitative methodology for our study because it is very appropriate when one wants to fully understand a complex subject (lack of BP control among patients receiving free BP treatment), from the perspective of the people who experience the phenomenon [27, 28]. Our constructivist perspective inclined us to believe that there are Botswana context factors that affect BP control among treated patients in Botswana. At the time of our study, no qualitative study had been done to solely explore barriers and facilitators of BP control among treated patients in Botswana.

## 2 Methodology

Interviews were carried out by Irene Kedisang (I.K.), a middle‐aged female who is trained in qualitative and quantitative research. She has received research ethics training, Framework for Optimization, Quantification of Uncertainty, and Surrogates (FOQUS) training, Population Services International (PSI) training, Dashboard Data Analysis training and has previously worked for PSI (Botswana), as well as the Botswana Family Welfare Association. She has vast experience in data collection, processing and analysis.

The authors of this article were V.S. (male family physician—MD, MBA, MFamMed, FGHL and PhD), Y.B. (female, family physician—MBBS and MMed), N.Y. (female, clinical project manager—BSc, MSc and PhD) and J.M. (male, cardiologist—MD, MMed, MSc in cardiology and MSc in clinical epidemiology). All authors had more than 5 years of experience in their disciplines and in research at the time of the study.

V.S., Y.B. and J.M. were familiar with the public PHC clinic environment because of their work in these clinics. They, however, did not have personal knowledge of the participants and did not meet them during the study. I.K. was familiar with the public PHC clinic environment which she sometimes patronised. She, however, did not have prior personal knowledge of participants. I.K. was told not to disclose the identities of the researchers to participants, to avoid participant bias.

Because of their familiarity with the public PHC clinic environment and patients, V.S., Y.B. and J.M. were conscious of their biases and tried to bracket them during data analysis and when writing the manuscript. N.Y. did not have any significant biases to bracket because she did not work in the public PHC environment.

**The conceptual framework** of this study included that qualitative research helps us to understand in depth, complex phenomena (in this case the lack of BP control in patients treated for BP, who receive free healthcare) [29]. We used a descriptive qualitative research study approach because we wanted to describe the problem only, in the way that the participants perceived it, as completely as possible, with minimum interpretation by the authors [30].

This was a qualitative study that explored the barriers to BP control among patients receiving BP treatment in public clinics in Gaborone, Botswana. Audiotaped structured interviews were used to collect data. V.S., the principal investigator (PI), is trained and experienced in qualitative research and has qualitative research publications to his name. From the literature, V.S. noted the topics that affected BP control in patients who were on BP treatment. V.S. included these topics in the interview guide, and he also used his experience as a family physician with more than 30 years of experience in treating BP patients to make the interview guide relevant to the experiences of Botswana BP patients and the health system that served them. This interview guide was piloted among some patients, nurses, doctors and district health managers. From the pilot study, the interview guide was adjusted to make it more understandable and to deliver data that was pertinent to the study. Patients, nurses and doctors were recruited from two large public PHC clinics at opposite ends of Gaborone, whereas district health managers were recruited from their offices in Gaborone. The Ministry of Health (MoH) in Botswana has divided the country into health districts. District Health Management Teams (DHMTs) implement MoH health policies and deliver free health services to 98% of the population. The Botswana health system comprises a university teaching hospital, six tertiary hospitals (one public), many public district and primary hospitals, and many public and private PHC clinics. Most patients get health services from public PHC clinics, through consultations with doctors and consulting nurses. Patients with high BP visit public clinics for assessment and prescriptions at regular intervals that depend on how well their BP is controlled.

Sampling was purposive such that participants were adult BP patients on treatment, nurses who consult with patients, doctors and district health managers. Sampling also ensured diversity in BP treatment experience, gender and age. Sample size was guided by data saturation [31]. Recruitment started in April 2023 and continued for 6 months, to September 2023. Only adult (≥21 years of age) patients, nurses, doctors and district health managers of a sound mind, who gave their consent to participate were recruited.

Consenting participants were included in the study after full explanation of the research, after participants’ mode of participation and ethical issues regarding their participation were explained. One research assistant (RA) collected data in English or Setswana. The RA had received prior training in qualitative methods, including interviewing and research ethics. The RA introduced herself to the clinic manager and showed her research ethical clearance certificates from the University of Botswana (UBR/RES/IRB/BIO/343), the MoH (HPRD:6/14/1) and the DHMT [GGDHMT 6/17/1 III (39)].

All interviews were face to face. At the beginning of an interview, the RA introduced herself and greeted participants in a culturally appropriate manner before engaging them about the study. Before interviewing a participant, the RA explained the research to the participant and made him aware of his rights as a free person to accept or decline participation or stop participating in the study at any time. The RA addressed all concerns that a participant had before asking the participant to sign a consent form. Thereafter, the participant signed two consent forms: to be interviewed and for the interview to be audio recorded. The RA then proceeded to interview the participant.

Eighteen participants (six patients, four nurses, four doctors and four district health administrators) participated in the study. A few patients declined to participate citing other urgent commitments like employment as reasons.

Patient interviews were done in private, in the clinic, in a room provided by the clinic manager. Nurse and doctor interviews were done in private, in the consultation room when there were no more patients to consult. Where this was not possible, doctors were interviewed in private in a quiet restaurant. The interviews occurred from Monday to Friday, during mornings and afternoons.

The RA asked open‐ended questions in an iterative manner, reflected to participants what she understood the participant to be saying, sought clarification, asked one question at a time, asked short questions and probed (asked follow‐up questions), to get deep data. She had two audio recorders, in case the recorder she was using failed. The RA also collected demographic data in a form and wrote field notes about her study experiences in a journal. The interviews were made anonymous by identifying participants’ interviews by numbers. The interviews were loaded into a password‐locked laptop, and the audio recorders were placed in a safe place, which was lockable.

The RA conducted an interview until the RA felt there was no new information from the participant (data saturation) or until the participant desired to stop being interviewed. Study interviews were stopped when no new data were coming out of the interviews (until data saturation). For this study, interviewees were viewed as one group that was located in the public PHC context. For the sake of good representation of perceptions on the topic, purposive selection ensured inclusion of patients, nurses, doctors and district health managers.

For the initial three interviews, the RA and the PI listened to the audio recordings of interviews together, and the PI assessed the quality of the interview; whether the RA probed, sought clarity, reflected back to the participant, was iterative enough in questioning, asked open‐ended questions and followed the interview guide. The RA and the PI also assessed the user‐friendliness and the clarity of the interview guide to participants and the RA. The technical quality of the audio recordings was also checked. The interview guide and the interview process were discussed to see if there were areas needing improvement, and remedial action was taken as needed.

As she interviewed participants, the RA became aware of the main ideas (codes/themes) that participants were repeating, and as the audio recordings were passed on to him, the PI listened to them and got an impression of the main ideas from the interviews. After data collection completion, the interviews were transcribed by the RA. The RA transcribed the local language audio recordings in the local language. The local language transcripts were checked for accuracy by V.S. using the local language audio recordings. The local language transcripts were then translated into English by the RA and V.S. checked the accuracy of the translations. The RA transcribed the English interviews, and the transcripts were checked for accuracy against the audio recordings by V.S.

Data were analysed for themes using a method adapted from a process described by Braun and Clarke [33], Table 1. Both the V.S. and the RA were proficient in English and the local language. V.S. and Y.B. (both family physicians) were familiar with the clinics and the type of patients taking BP medication at these clinics. V.S. and Y.B. were also familiar with the administrative structure of DHMTs. V.S., Y.B. and N.Y. read the English language transcripts several times. Three similar transcripts were then coded independently by V.S., Y.B. and N.Y.

**TABLE 1:** Data analysis steps.

Serial number	Action
1	V.S. listened to audio recordings several times. This gave him an idea of commonalities in what participants said
2	RA transcribed audio tapes in the language in which interviews were conducted
3	V.S. checked transcripts for accuracy by reading them while listening to audiotapes
4	RA translated local language transcripts into English
5	V.S. checked translated transcripts for accuracy by reading them while listening to local language audiotapes
6	V.S., Y.B. and N.Y. coded the same three transcripts which were chosen randomly. They coded these independently
7	V.S., Y.B. and N.Y. compared codes and discussed them until a consensus list of codes was made
8	V.S iteratively grouped and regrouped codes several times to derive themes from the codes. This resulted in the production of a codebook
9	V.S. showed the codebook to Y.B. and N.Y. for discussion and consensus on the final codebook
10	The consensus code book was uploaded into NVivo and used to code the rest of the 18 transcripts. V.S. did the NVivo coding
11	V.S. came across a few new codes during NVivo coding, and he discussed these with Y.B. and N.Y. and allocated the codes to existing themes, or new themes were created as in Actions 8 and 9

Abbreviation: RA, research assistant.

V.S., Y.B. and N.Y. discussed the codes they independently came up with until consensus was reached on the codes to be used in analysing the transcripts. Using the codes that were agreed upon, V.S. created a code book by iterative grouping and regrouping of codes. The codebook comprised themes and subthemes with their definitions. The code book and the transcripts were uploaded into NVivo software [34]. The codebook was used to analyse the transcripts in Nvivo.

## 3 Results

There were 18 adult participants in the study. They consisted of six patients, four nurses, four doctors and four district health managers of different BP treatment experience, gender and age. A structured interview was conducted with each participant, and the shortest interview was 18 min, whereas the longest lasted for 43 min. The average time of the interviews was 33 min.

The barriers to BP control were health system‐related, patient‐related, medicine shortage‐related and lack of knowledge‐related. There were also outlier themes (silent killer and use of other medicines), which only two patients talked about.

### 3.1 Health System‐Related Barriers

These barriers seemed to emanate from the processes and structure of the health system.

*Facility‐related* barriers to BP control were clinic factors that constrained BP control. These included having *too many patients* at the clinic, which discouraged patients from coming to the clinic.

‘When I come to the hospital (clinic) and there is a lot of people like this it also makes me to stop (coming).’, said patient B902.

*Shortage of staff* was a barrier that described the shortage of nurses and doctors in the clinics. Because of this shortage, doctors moved from one clinic to another.

‘Patients’ turn‐up at the clinics is too high and you would find that in Botswana just as is, there is only one doctor! And one as he would be, he (the doctor) would spend (few) hours in this clinic and would go and spend (few) hours in that (another) clinic.’, said district health manager DHMe.

‘Yes, really it's just that workload is too much, so there is shortage of staff regarding nurses.’ said district health manager DHMn.

*Long waiting times* Patients waited for long periods to be consulted by a doctor or nurse. This caused patients to suffer a lot of stress.

‘That is now what is making BP to go high. Yes, isn't it that you can become angry? You'll be seeing that it's slow like today, do you know that we've been long here (here for a long time)?’, said patient BR9.

*Loss of records* was a barrier that described loss of patients’ personal files, laboratory results and x‐rays due to lack of adequate use of electronic medical records. When patients lost their personal medical files, and when the clinic could not find laboratory reports or x‐rays, this made it difficult to keep track of patients’ BP control, medications and health outcomes.

‘you may attend to somebody and you are not able to realize that they were not being controlled over time because cards are not left behind (in the facility) they (patients) go with them. So when they come they may not bring them……we have not computerized, and the data is not remaining in the facility.’, said district health manager DHMcur.

*Lack of funds, and BP medication* described underfunding of the healthcare system as a barrier to BP control.

‘It has been sporadic (drug supply); I would say sporadic; sometimes it is there and sometimes is not there. Problem is that there are just too many items that are not available.’, said district health manager DHMp.

The shortage of discretionary funds that pharmacists could use to buy drugs that were out of stock was frustrating.

‘Especially when it comes to things that are not available, …. you are limited by the ceiling; the amount of money that you can use at one point.’, according to district health manager DHMp.

‘I'm assuming everything right now is due to the financial situation of the country, emmm… that's why not only the BP medications are out of stock, but a lot of other things are out of stock.’, said Doctor07.

*Shortage of BP machines, shortage of correct BP machine cuffs and lack of regular recalibration of BP machines* was another barrier that impinged on optimum control of BP.

‘… we have BP machines…in our facilities; …we do have but it is not enough….we can be given such money for procurement, we are able to order but we don't have enough; I mean we wish we could be having more than what we have….’ said district health manager DHMn.

‘…. we just have one blood pressure cuff which is… I think the medium size, it should give an average representation for most patients…But if (a) patient is obese for example, it will overestimate the readings…. and if the patient is underweight, you'll get them a lower blood pressure reading’, said Doctor18.

Patient MO20 said, ‘And the issue of equipment mostly, it's the issue of cuffs and BP machines that are never taken to “Mams” for calibration.’

### 3.2 HCW‐Related Barriers

These barriers had to do with the attitudes, behaviours and knowledge of HCWs.

*Staff attitudes* were barriers that inhibited doctors’ and nurses’ interactions with patients. Negative staff attitudes contributed to lack of BP control.

‘Of course, if the doctors and the nurses shout at you, they contribute for BP to be high.’, said patient B902.

*Inadequate counselling of patients and lack of follow up:* This barrier described the lack of patient education about lifestyle, medications and the importance of patients keeping clinical review appointments. This contributed to lack of BP control.

‘And with hypertension we just prescribe then they go but if they do miss (a review appointment), we seldom call the (patient)…there is strict adherence counselling for HIV medication but there is lesser strict adherence counselling with hypertension.’, said district health manager DHMcur.

‘Not all places, I think one or two clinics around, do have maybe counsellors.’, said patient MO07.

*Doctor's lack of knowledge* described doctors’ and nurses’ lack of adequate knowledge about the treatment of high BP. This lack of knowledge inhibited BP control.

Doctor 18 said, ‘because if one doctor is unaware of the best practice, they are emmm…going to… they are not going to carry forward the best possible treatment.’

### 3.3 Patient‐Related Barriers

These barriers pertained to the behaviours, lifestyles and socioeconomic conditions of patients.

Patient‐related barriers to BP control could be categorised as non‐adherence, lifestyle issues, social issues and stress.

*Non‐adherence* to treatment was a barrier to BP control.

‘…sometimes when you see someone's BP going high, sometimes it could be that he does not take doctor's instructions well, sometimes he would not be taking his pills properly.’, said patient BR09.

*Lifestyle issues* like inappropriate nutrition, drinking, smoking and lack of exercise, contributed to lack of control of BP.

‘I just think that really it's lifestyle mostly, I don't think these pills are failing.’, said patient BR12.

Doctor 19 Said, ‘So, Patients most of the time they have difficulties with keeping up with the lifestyle modifications.’

‘Others it would be that they drink alcohol. Isn't it that you'll find that when a person has gone out to have fun, sometimes he would have left pills back at home. When going for those ‘all nights’ (parties) obviously he would not be taking them.’, said nurse 005.

‘Do you understand what I am saying? Weight contributes’, said patient 02.

‘… so that poverty can also contribute to a (certain) lifestyle. Or even the problems that I have already alluded to, they can make one reach a point where now it (BP) becomes uncontrollable’, said district health manager DHems.

*Unfavourable socioeconomic issues* made it difficult to control BP.

The elderly had challenges which contributed to lack of control.

Nurse 14 said, ‘… for old people, some the vision is poor, they can't even remember the names of the medications, they can't even know when they are coming for review.’ The effects of aging, for example, vision and memory loss impaired the elderly's social function (not remembering the names of their medications if they were asked what medications they were taking, and not keeping review dates, because of loss of memory and vision).

Some patients were not able to buy BP medications when free BP medications were not available in the clinics.

‘I might be working but with my cash at hand I can't be able to buy for myself because BP pills are expensive.’, said patient 10.

*Stress* or anxiety was a common barrier to BP control.

Patient BRIII12 said, ‘It's almost month end. I have a child. I have to … to send him money and I haven't even found a piece job, and this is stressful’.

Patient BL9 said, ‘Like really the other thing that could be causing BP what I have realized like me, I am the kind of person who stresses… It's just that people with BP don't want to be hurt a lot’.

‘So whenever I was facing the entrance knowing that it's almost my time to go inside (to consult the doctor/nurse) then my heart would go thu…thu…thu (pumping very fast)’, said patient Br III 09.

## 4 Discussion

In our study the barriers to BP control among treated patients in public clinics in Gaborone, Botswana, were health system‐related, patient‐related and health worker‐related. These findings are like findings in previous studies [24, 35].

### 4.1 Health System‐Related Barriers

Health system‐related barriers to BP control in our study revealed that staff and drug shortages, BP machine shortages, non‐calibration of BP machines and use of a one‐size cuff for all BP patients compromised BP control. Our findings corroborate the conclusions of the American Heart Association and the American Medical Association which stated that staff shortages were a barrier to BP control [36]. A study in Ghana also mentioned staff shortages as a barrier [37]. Similarly, staff shortages as a barrier to BP control were like the findings of a study in Asmara, Eritrea, that mentioned patient overload as a barrier to BP control [38]. Patient overload may result from staff shortage.

Medication availability was the most common barrier in a systematic review and a meta‐analysis [39]. A study in Ethiopia [33], Ghana [37] and another one in Nepal [40] also mentioned drug shortages as a barrier to BP control. We did not find the difficulties of navigating the health system nor the cultural differences mentioned by Hispanic patients in Chicago, the United States [41].

Incorrect BP measurement (e.g., from use of a wrong‐size cuff) was a barrier to BP control in our study, and this corroborates a US study that stated that correct‐size cuffs are central to correct BP measurement [42]. We found that shortage of BP machines and their poor maintenance was a barrier to BP control, corroborating a study in Ugandan public PHC facilities which experienced shortages of BP machines and their poor maintenance [43].

We found that shortage of laboratory reagents hampered BP control. This finding corroborates findings in a thesis on laboratory medicine in Africa that found a number of challenges, including resource and supply chain challenges, that made African laboratories inadequate in supporting medical services [44].

### 4.2 HCW‐Related Barriers

Our study showed that HCW‐related barriers were negative HCW attitudes towards patients and the lack of counselling for patients about BP. Our findings corroborate the findings of three studies: one in urban Nepal; another in Asmara, Eritrea; and another in Chicago, Illinois [38, 41, 45] that found lack of counselling as a barrier to BP control. Counselling of patients in our context, in relation to BP control, meant educating patients about the chronicity of BP, the need for lifestyle changes and adherence to treatment, as well as keeping BP review appointments.

Poor HCW attitudes and poor communication were reported as barriers to BP control in a systematic review and meta‐analysis [39] and in a study in Tanzania [46]. A study in Colombia also showed that poor doctor attitudes inhibited BP control [47].

We also found that when doctors lacked knowledge about correct BP treatment or prescribed pharmacotherapy incorrectly, BP control was compromised. These findings corroborate findings in other studies that showed that treatment inertia and inadequate knowledge of BP treatment among HCWs inhibited BP control [24, 25, 35].

### 4.3 Patient‐Related Barriers

Certain behaviours, lifestyle and socioeconomic conditions of patients compromised BP control. Non‐adherence to medication, alcohol intake, old age and feeling well despite having a high BP were some of the inhibitors of BP control in our study. This corroborates the findings of studies in Eritrea and Ethiopia [21, 38]. Unhealthy lifestyles like drinking alcohol, smoking, eating unhealthy diets and lack of exercise were also found to be barriers to BP control in a study in Colombia, where patients found it difficult to adopt healthy lifestyles [47], and in a systematic review and meta‐analysis [39].

We found that stress and other unfavourable social issues like old age and poverty contributed to lack of control of BP. A study in Singapore showed that older age and being retired or unemployed were barriers to BP control [48]. A study in Switzerland PHC settings revealed that older age, increased BMI and smoking were barriers to BP control [49]. A systematic review also indicated that old age is a barrier to BP control [35]. In our study, stress was reported to be a barrier to BP control, and there is good evidence that stress elevates BP (e.g., white coat hypertension and other stress‐inducing phenomena) [50]. It is generally believed that stress increases BP by stimulating the sympathetic‐adrenal system [51].

### 4.4 The Importance of This Study

The importance of this study is that it is the first of its kind in Botswana that focused solely on exploring the barriers to BP control, as far as we know. This study also indicates which barriers to BP control are most relevant to the Botswana setting. These are therefore the barriers that need to be overcome in all efforts to improve BP control among treated patients in Botswana. As implementation science has shown, EBIs need to be relevant to stakeholders and the environment in which they are applied [52]. This is why it was important to include patients, HCWs and health managers to understand this phenomenon (uncontrolled BP in treated patients). We hope that the findings of this study present credible evidence for crafting interventions to improve BP control among treated patients.

### 4.5 Recommendations From This Study

- Adequate health funding and prudent use of health funds should alleviate shortages.
- Good management at the central government, district and clinic levels should alleviate supply chain challenges and facilitate appropriate staffing and stocking levels.
- Performance incentives and regular knowledge evaluation may help HCWs to continuously upgrade their knowledge and practice.
- Some clinic staff should be dedicated to educating patients about BP, the need for adherence, lifestyle change and the importance of keeping BP review appointments.
- Socioeconomic challenges (old age, poverty, frailty, living alone and illiteracy) can be addressed through social programmes that address these challenges.
- The elderly can be helped with taking medications as prescribed and not missing BP review appointments.
- Poverty alleviation programmes should also address medicine access for the poor. The extended family should be involved in all phases of BP care for patients who need social support.

### 4.6 Study Limitations

This was a qualitative research study, and its findings reflect the perceptions of the participants. Though its findings corroborate the findings of other studies, its findings cannot be generalised, though qualitative researchers would argue that the findings are transferable to similar settings with similar participants. A follow‐up quantitative study will be done to assess the validity of these findings across Botswana, among patients, nurses, doctors and health managers.

In this study, we did not treat patients, nurses, doctors and district health managers as separate entities, but as members of the PHC ecosystem, just as one would explore the perceptions of women without seeking to differentiate the perceptions of lesbians, straight and trans women. It would be useful to do a follow‐up study to explore and compare the perceptions of patients, nurses, doctors and district health administrators. This may highlight different understandings of the lack of BP control among treated patients and inform interventions in a more sophisticated manner.

### 4.7 Stakeholders and Roles of PI and RA

This study involved patients, nurses, doctors, clinic managers and district managers. To gain access to the clinics and the DHMT staff, we had to get approval from the DHMT, and the head of the DHMT was instrumental in getting us this approval. Likewise, we needed the approval of the clinic managers to gain access to patients, nurses and doctors who were under their management. Both the PI and the RA were involved in negotiations to get these approvals.

Both clinic and DHMT staff were interviewed in this study. However, we do not know if the DHMT head or clinic managers influenced the interviews that were conducted. All the interviews were conducted in private.

The PI had clinic duties at the study clinics and knew the DHMT head. He, therefore, did not conduct any interviews, and the RA was instructed not to tell participants of his involvement in the study. The RA was trained in qualitative study interviewing before the study to minimise her influence on the data.

### 4.8 Trust Worthiness of the Study

The trustworthiness of the study was ensured by the fact that two of the authors and RA knew the context of the study well. Though they may not have known the participants at a personal level, they came from the same community, spoke the same language, and worked in or used the same health system. Data were collected by audio recorders to ensure its veracity. The RA supplemented audio recordings with pertinent data that she collected in her field journal. Validated interview guides were used to collect data that were relevant to the research objectives. Three analysts initially, independently, developed codes that were later discussed among the three, and a consensus was reached as to what codes were to be used to collect the data. Bracketing was used to minimise the biases of each analyst during the initial coding and throughout the rest of the coding process. The well‐known coding process described by Braun and Clarke was followed in analysing the data. The results of the study were presented to participants in seminars and accepted.

## 5 Conclusion

This study revealed that barriers to BP control among patients who were on BP treatment BP‐included health system factors, HCW‐related factors and patient‐related factors. Resource shortages, HCW lack of knowledge and attitudes, lack of adherence to prescriptions, lifestyle issues and socioeconomic factors were among the more common barriers to BP control. These barriers seem to be rooted in inadequate health funding, HCW and patient education, healthcare management and lack of social support for the poor and elderly. Attending to these barriers may help decrease the high prevalence of uncontrolled BP among patients attending public PHC clinics in Gaborone, Botswana.

## Author Contributions

V. Setlhare, N. Youssouf and J. Mwita were involved in the conceptualisation of the study, and all authors reviewed the manuscript. V. Setlhare, N. Youssouf and Y. Bogatsu analysed the data, and V. Setlhare wrote the manuscript.

## Conflicts of Interest

The authors declare no conflicts of interest.

## Data Availability

All data concerning this study will be made available on reasonable request to the corresponding author.
